# Accessing forgotten memory traces from long-term memory via visual movements

**DOI:** 10.3389/fnhum.2014.00930

**Published:** 2014-11-18

**Authors:** Estela Càmara, Lluís Fuentemilla

**Affiliations:** ^1^Cognition and Brain Plasticity Unit, Institute of Biomedical Research of Bellvitge (IDIBELL)Barcelona, Spain; ^2^Department of Basic Psychology, University of BarcelonaBarcelona, Spain

**Keywords:** long-term memory, eye movements, forgetting, associative memory, implicit memory

## Abstract

Because memory retrieval often requires overt responses, it is difficult to determine to what extend forgetting occurs as a problem in explicit accessing of long-term memory traces. In this study, we used eye-tracking measures in combination with a behavioral task that favored high forgetting rates to investigate the existence of memory traces from long-term memory in spite of failure in accessing them consciously. In two experiments, participants were encouraged to encode a large set of sound-picture-location associations. In a later test, sounds were presented and participants were instructed to visually scan, before a verbal memory report, for the correct location of the associated pictures in an empty screen. We found the reactivation of associated memories by sound cues at test biased oculomotor behavior towards locations congruent with memory representations, even when participants failed to consciously provide a memory report of it. These findings reveal the emergence of a memory-guided behavior that can be used to map internal representations of forgotten memories from long-term memory.

## Introduction

It is commonly agreed that forgetting can be characterized by an apparent loss of information already encoded and stored in an individual’s long-term memory (Decay theory (Woodworth, 1938); Consolidation theory (Dudai, 2004)) or by a process in which old memories are unable to be retrieved from memory storage (Interference theory (Underwood, 1957); Retrieval failure theory (Tulving and Thomson, 1973)). Yet, disentangling between these two is not trivial. Because retrieval often requires a conscious response, it is difficult to determine whether the eventual inability to recollect memory information does actually represent a complete or partial vanishing of it or instead, it appears as a problem in accessing consciously the selective memory trace. Thus, it is of significance to find sensitive measures of memory that could provide indexes of the existence of memory traces independently of overt responses.

Recent studies in humans indicate that eye movements can reveal memory for elements of previous experience, even without appealing to verbal reports and without requiring conscious recollection (Hannula et al., 2010). These effects rest on the observation that eye movements are biased towards concurrent visual input matching or mismatching the information encoded in past episodes (Ryan et al., 2000, 2007; Hannula et al., 2007; Hannula and Ranganath, 2009). However, because these experimental settings are characterized by an at least partial display of visual information during memory testing, the question of whether and to what extent any effects in eye movement behavior are purely guided by internal memory representation (Ferreira et al., 2008), by externally-guided visual stimulation triggering memory reactivation (Richardson et al., 2009) or both, in the absence of awareness remains elusive.

To address questions about whether or not gaze is attracted to locations (i.e., indexing a spatial memory trace) that had previously been occupied by studied content when blank screens were presented at test, Spivey and colleagues studied eye movement patterns when participants visually scan a blank screen while a memory cue is provided. Indeed, participants’ encoding of spatial information was revealed by their looking behavior when answering a question that related to information that had previously been presented in an empty region of space (Richardson and Spivey, 2000; Spivey and Geng, 2001). These experiments showed that even in front of a completely blank grid, participants would make systematic saccades to the region of space where they perceived the event. This suggests that there might be an aspect of memory below the level of explicit awareness that could be dissociated from retrieval operations. However, these experimental findings were accounted for in circumstances in which memory for spatial location was not tested directly (via explicit report), and therefore do not address questions about whether or not memory for location was evident in eye movement behavior absent explicit awareness. Thus, this methodological aspect hampered the possibility to know whether eye movement behavior represents a sensitive measure of memory that could provide indexes of the existence of memory traces independently of overt responses.

**Figure 1:**
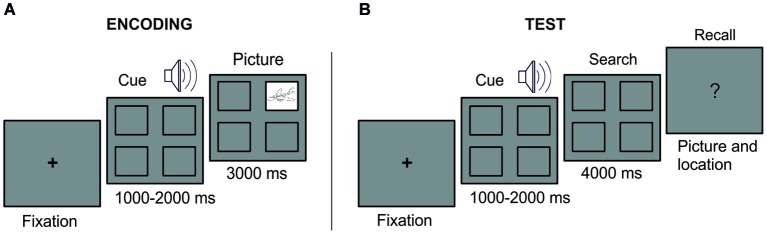
**Experimental Design**. At encoding **(A)**, a fixation cross remained in the center of the screen until eye fixation. A sound cue was presented with four empty squares at the screen. At the end of the sound cue, a picture appeared in one of the squares during 3 s. A complete empty screen of 2.5–3.5 s duration separated the start of the next trial. At test **(B)**, after a fixation cross, each sound cue was presented with the four empty squares on the screen. At the end of the sound cue, the searching period started. Participants were instructed thereafter to verbally report the associated picture or to indicate “no memory” when the information was forgotten.

In the current study, we sought to overcome these limitations with the use of eye-tracking measures in combination with a new experimental approach. We designed a behavioral task in which unique sound-picture-location associations were presented once during an encoding phase. Critically, we set a large amount of associations during encoding in order to impoverish their conscious recollection in a later memory test, thus resembling conditions of severe memory forgetting, accompanying for instance certain type of clinical and neurological population (i.e., patients with brain lesions in the medial temporal lobe). At test, each sound was presented briefly and participants were instructed to visually search in the empty scan for the correct location of the associated picture (see Figure 1). Each trial finished with a verbal report whether or not they remembered the location (Experiment 1) and a confidence judgment about the memory for the object location (Experiment 2). Drawing on the idea that oculomotor behavior represents a reliable index of memory access of long-term memory, we expect that sound cues at test would trigger a memory reactivation of the associated visual information that could emerge as a biased pattern of eye movement towards space locations congruent with memory trace representation, even for those trials in which participants failed to consciously provide a memory report of it.

## Materials and methods

### Ethics statement

All participants provided written informed consent at the beginning of the experiment, and were provided with a written debrief form after the experiment. All procedures were approved by the local ethics committee (University of Barcelona). All participants were compensated with credit courses for their participation.

### Experiment 1

#### Participants

Twenty participants (12 women, *M* = 20.2 years, SD = 1.1) took part in Experiment 1. All participants were students from the University of Barcelona. Four of them were excluded from the analysis because of technical problems with eye movement recording. Participants were with no history of neurological or psychiatric episodes, and had normal visual acuity.

#### Stimuli

Stimuli consisted of 44 not semantically-related pairs of pictures and sounds that were randomly selected for each participant. Pictures were black-and-white line drawings, selected from a drawing database executed according to a set of rules that provide consistency of pictorial representation (Snodgrass and Vanderwart, 1980). The pictures have been standardized on four variables of central relevance to memory and cognitive processing: name agreement, image agreement, familiarity, and visual complexity. All 44 auditory cues were natural sounds extracted from a database provided by the Spanish Ministry of Education, Culture and Sports^1^. The sounds were all easily recognizable (based on a pilot study with healthy participants; *n* = 6) and had a duration ranging from 1 to 2 s.

#### Behavioral task

The paradigm consisted in an encoding and a test phase (see Figure 1). During the encoding phase, we encouraged participants to learn 44 different associations of sounds cueing pictures, each appearing in a specific square of the screen (the two initial and the two last associations of the list served as primacy and recency effect buffers, and were not examined at test). Pictures were equally distributed in the four possible locations and presented randomly and counterbalanced for each participant. Participants were informed before the encoding phase that each picture-sound-location was presented only once and that a test would follow and that they would be required to indicate whether they remembered the location and the picture. At encoding, a fixation cross remained in the center of the screen until eye fixation. A sound cue was presented with four empty squares at the screen. At the end of the sound cue, a picture appeared in one of the squares during 3 s. A complete empty screen of 2.5–3.5 s duration separated the start of the next trial (i.e., indicated by the appearance of the fixation cross). At test, each sound cue was presented and participants were asked, during a subsequent “search period” of 4 s, to fixate their viewing to the quadrant in which picture appeared at encoding. In case they could not retrieve the picture location, they were told to visually scan the monitor as if they were searching for the correct picture location. They were told that such searching behavior could be helpful to retrieve the memory information. First, participants answered with “yes or no” their recollection of the picture location and then whether they could retrieve the picture itself. In such case, they were further asked to name the picture object. To minimize as much as possible any verbal representation of picture location (e.g., labelling upper-left corner as “one”, upper-right corner as “two” and so on), participants were not further asked to report it. Once participant reported the verbal response, the experimenter manually (i.e., by pressing the space bar) initiated the start of the next trial. The order of the trial appearance was randomized during the both the study phase and the test phase.

#### Procedure

Stimuli were displayed on a black background on a 21” CRT monitor (1024 × 768 pixels, refresh rate 150 Hz) with approximately 9° of visual angle, corresponding to square images of 9.5 cm at a viewing distance, using the Psychophysics toolbox extensions for Matlab^2^. The participants were seated with their eyes approximately 60 cm from the computer screen with powerful speakers in a dimly illuminated testing room.

Eye position was monitored at 500 Hz using an EyeLink II head-mounted eye tracker (SR Research). Oculomotor data were parsed into saccades and fixations using Eyelink’s standard parser configuration, which classifies an eye movement as a saccade when it exceeds 30°/s velocity or 8.000°/s^2^ acceleration and amplitude of gaze shift was a minimum of 0.15°. The endpoints of saccades were determined with respect to whether they fell within any of the four quadrant of stimulus presented on the screen.

Oculomotor memory-guided behavior was indexed by the fixation (the relative number of fixations) and the dwell time proportion (the relative time during which the gaze remains fixated) on the correct square during the search period. This analysis was performed using customized Matlab code (The Mathworks, Inc., Natick, MA, USA) for each trial and then results were sorted as those followed by a verbal report of having remembered the location (recollected trials) or not (forgotten trials), independently of whether picture labels could be verbally retrieved.

From a purely random behavior it would be expected the average proportion of eye movement measures to each location follow a binomial distribution with *p* = 0.25. Given that the number of trials is large, the binomial distribution can be approximated with a normal distribution of mean 25%. Measures significantly over this value in a Student *t*-test were considered as evidencing the reactivation of memory representations of picture location. Statistical significance in all the tests was set at *p* < 0.05, one-tailed. Significant threshold was adjusted with Bonferroni correction for multiple comparisons when more than two comparisons were made with the same measure. Measures of effects size (Cohen’s *d* or *f*) were also provided. SPSS (SPSS Inc., Chicago, USA) software was used for the statistical analysis.

#### Results

**Figure 2:**
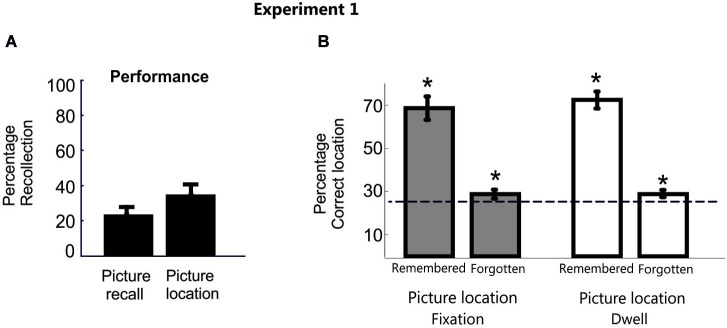
**Behavioral data in Experiment 1**. **(A)** Percentage of correct picture and location recall responses averaged across participants for Experiment 1. **(B)** Bar plots represent the proportion of fixation and dwell time in the correct picture location averaged across participants in Experiment 1. Error bars denote Standard Error of the Mean. * *p* < 0.05; “n.s.” denotes *p* > 0.05.

Overall, the proportion of trials by which participants reported correctly the picture label (*M* = 24.7%, SD = 17) or indicated they had successfully retrieved the target location (i.e., a “yes” response) (*M* = 36.7%, SD = 17.6%) was very low (Figure 2A). As expected though for both conscious recollection of picture and location, participants’ visual movements were strongly, albeit not exclusively, distributed in the correct square location (fixation proportion: *M* = 70.1%, SD = 23.8%; dwell proportion: *M* = 72.5%, SD = 18.4%). The possibility that participants’ needed some time to fully recover consciously the correct memory during the delay period may partially explain why the pattern of eye movements was not distributed 100% in the correct location in those cases. Another possibility is that the inherent exploratory behavior of eye movements induced shifts of eye movements throughout the screen during such long time period.

However, a disproportionate eye movement pattern towards the correct location was also shown during the search period in those trials whose position participants explicitly reported to have forgotten, independently of whether the object recall was correct or not (Mean fixation proportion = 30.02%, SD = 6.1%, *t*_(15)_ = 3.3, *p* < 0.001, *d* = 1.2; Mean dwell time = 30.5%, SD = 5.7%, *t*_(15)_ = 3.8, *p* < 0.001, *d* = 1.4). Importantly, these results were consistent even when excluding from the analysis those trials that participants were able to label verbally the picture object but not its location (Mean fixation proportion = 28.9%, SD = 7.1%, *t*_(15)_ = 2.15, *p* = 0.01,* d* = 0.8; Mean dwell time = 29.4%, SD = 7%, *t*_(15)_ = 2.48, *p* < 0.01,* d* = 0.9; Figure 2B).

### Experiment 2

The aim of Experiment 2 was to address the question of whether the awareness test in Experiment 1 based on a “Yes/No” answer could be insufficiently sensitive to failures in memory access. Thus, it could be argued that on a subset of the trials participants felt that they may know the location, but were not confident enough to indicate that they had successfully recalled it. If this were the case, then viewing effects reported in Experiment 1 when explicit recall had “failed” may actually reflect conscious access to information about sound-location associations.

#### Participants

A new sample of 20 participants (17 women, *M* = 23 years, SD = 4) took part in Experiment 2. All participants were students from the University of Barcelona. Participants had no history of neurological or psychiatric episodes, and had normal visual acuity.

#### Procedure

The same stimuli, apparatus and behavioral task as in Experiment 1 were used, except that participants were instructed to provide their confidence about the memory of the location at the end of each trial during the recognition phase. Thus, just after the “search period” a message appeared on the screen requesting the participants to report whether their memory for the location of the picture in such trial was “100% forgotten // 50% forgotten // 50% remembered // 100% remembered”. In this way, we were allowed to analyze separately those trials in which participants reported to be completely sure they have forgotten the picture location (100% forgotten) and those trials that despite participants had no access to picture location they could have some sort of feeling of familiarity about which could be the location of the picture (50% forgotten). These options were differentiated from those in which participants claimed that picture location was poorly accessible (50% remembered) but they had the feeling they may do a good job if they had to select between only two options (instead of the four possible locations) and those trials in which participants actually remembered the picture location (100% remembered).

#### Data analysis

Data analysis was the same as in Experiment 1 except that fixation and dwell time proportion on the correct square during the search period was analyzed according to participants’ confidence judgment of having remembered the picture location.

#### Results

**Figure 3:**
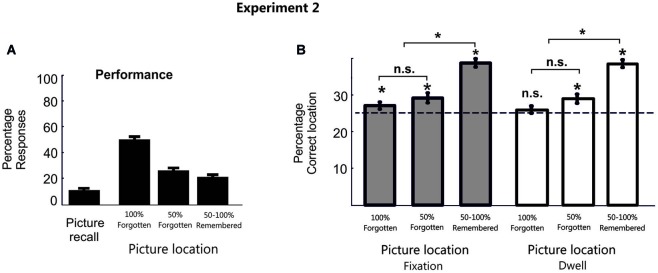
**Behavioral data in Experiment 2**. **(A)** Percentage behavioral responses averaged across participants for Experiment 2. **(B)** Bar plots represent the proportion of fixation and dwell time in the correct picture location averaged across participants in Experiment 2. Error bars denote Standard Error of the Mean. * *p* < 0.05; “n.s.” denotes *p* > 0.05.

As in the previous experiment, in most cases during the test phase participants did not recall the picture object (*M* = 80.2%, SD = 8.7%, with correct picture recall: *M* = 10.5%, SD = 6.9%; and with an erroneous object labelling: *M* = 9.2%, SD = 8.9%). For those trials in which participants did not recall the picture object, the confidence level of the memories for the object position was very low (*M* = 51.7%, SE = 4.6%; 100% forgotten; *M* = 26.6%, SE = 3.2%; 50% forgotten; *M* = 21.7%, SE = 4.14%; 50%–100% remembered; Figure 3A).

Consistent with previous results, even when participants failed to consciously provide a memory report of it, we observe a significant eye fixation pattern towards the correct target location during the search period (100% forgotten condition: Mean Fixation proportion for the target location *M* = 27.08%, SD = 4.5%, *t*_(19)_ = 2.05, *p* < 0.05, *d* = 0.66; Mean Dwell Time *M* = 26.3%, SD = 4.5%, *t*_(19)_ = 1.31, *p* = 0.1; 50% forgotten condition: Mean Fixation proportion for the target location *M* = 29.23%, SD = 6.1%, *t*_(19)_ = 3.1, *p* < 0.01, *d* = 1.1; Mean Dwell Time *M* = 29.2, SD = 6.1, *t*_(19)_ = 3.1, *p* < 0.01, *d* = 1.01). This is, when participants explicitly report to forget the location of the object (100% and 50% forgotten conditions) eye movements (especially proportion of fixations) showed a significant pattern towards the correct location. The lesser sensitivity of Dwell Time measures to detect patterns of memory reactivation in the 100% forgotten condition could be partially explained because it has been shown that search efficiency, or the overall time needed to find the target, is much more closely correlated with the number of fixations than to dwell times (Zelinsky, 1996; Zelinsky and Sheinberg, 1997).

Additionally, a repeated measure analysis (ANOVA) of eye movement patterns for the target location at each confidence was calculated. Two participants were removed from the 50%–100% remembered condition because they did not present responses at this confidence level. This ANOVA yielded a main effect of confidence (Mean proportion of fixations, *F*_(2,34)_ = 32.7, *p* < 0.001, *f* = 1.4; Mean Dwell time, *F*_(2,34)_ = 34.8, *p* < 0.001, *f* = 1.4). Interestingly, this effect showed both a significant linear (Proportion of fixation, *F*_(1,17)_ = 81.9, *p* < 0.001, *f* = 2.2; Dwell Time, *F*_(1,17)_ = 82.3, *p* < 0.001, *f* = 2.2) and a quadratic (Proportion of fixation, *F*_(1,17)_ = 6.68, *p* = 0.019, *f* = 0.6; Dwell Time, *F*_(1,17)_ = 5.9, *p* = 0.026, *f* = 0.6) trend, thereby suggesting that differences between confidence levels may not be totally proportional across them. In fact, paired Student *t*-tests (two-tail) comparing the different confidence levels confirmed significant differences (after correction for multiple comparisons, only *p-values* under 0.016 can be considered statistically significant) between the remembered and the forgotten conditions for the eye movement pattern (100% forgotten vs. 50%–100% remembered Mean proportion of fixation (*t*_(17)_ = −9.06, *p* < 0.001, *d* = −3.1), Mean Dwell time (*t*_(17)_ = −9.07, *p* < 0.001, *d* = −3.1); 50% forgotten vs. 50%–100% remembered: Mean proportion of fixation (*t*_(17)_ = −6.7, *p* < 0.001, *d* = −2.3), Mean Dwell time (*t*_(17)_ = −6.8, *p* < 0.001, *d* = −2.3), see Figure 3B). However, there were no significant differences within the 100% and the 50% forgotten condition (Proportion of fixation, *t*_(17)_ = 1.01, *p* = 0.33, *d* = 0.34; Dwell Time, *t*_(17)_ = 1.36, *p* = 0.19, *d* = 0.47), thereby excluding the possibility that the observed effect towards the target location can be interpreted only as differences in confidence level.

Finally, in order to rule out the possibility that the observed memory-guided eye movement patterns could be explained as a bias to eye movement preferences to specific locations, we tested whether target positions were equally distributed across the four positions for each condition in our participant’s performance. A one-factor (4 quadrant position) ANOVA indicated that the proportion of recalled location did not differ among the quadrants for any of the confidence conditions (“100% forgotten”: *F*_(3,57)_ = 1.9, *p* = 0.14, *f* = 0.32; “50% forgotten” : *F*_(3,57)_ = 1.7, *p* = 0.18; *f* = 0.3; “50–100% remembered”: *F*_(3,57)_ = 1.7, *p* = 0.17, *f* = 0.3), thereby discarding a bias in eye movement patterns for a preferred location.

## Discussion

In this study we used eye-tracking measures in combination with a new experimental approach to test the idea that oculomotor behavior may represent a reliable index of the existence of memory traces from long-term memory in spite of failure in accessing them consciously. Our findings show that the reactivation of associated memories by sound cues at test biased oculomotor behavior towards locations congruent with memory representations, even when participants failed to consciously provide a memory report of it.

Past studies have emphasized the implicit nature of eye movement patterns in recognition memory tests. Eye movements have been found to reflect previous exposure even in the absence of explicit awareness of the change (Althoff and Cohen, 1999; Hannula and Ranganath, 2009), and regardless of whether the task required intentional retrieval (Hannula et al., 2007). In fact, differential viewing of studied stimuli can be observed well in advance of explicit identification of that stimulus (Hannula et al., 2012). The present study is consistent with these past results in suggesting that eye movements provide an important sensitive measure of memory and expand them by showing that eye movement patterns are even biased towards memory content when this is reactivated by a non-visual associative cue.

Current and previous research provides experimental evidence that memory functioning can be tested reliably with the study of eye movements without the need to rely on conscious responses. Thus, patterns of eye movement varied according to the degree of how visual information matches/mismatches with existent long-term memory traces (Ryan et al., 2000; Smith et al., 2006; Hannula and Ranganath, 2009). Our findings add valuable information in tightening even more this link in indicating that, in fact, eye movement behavior can be guided by the internal memory representation without any concurrent input to the visual system. In Experiment 2, we further found that such memory-guided pattern of eye movements took place even in those cases in which participants reported confidently the information had vanished from long-term memory, thereby suggesting that eye movement behavior may act, at least partially, independently of subjective confidence of memory trace existence.

Despite that the current experimental design exploited the advantages of eye movement measuring to study implicit traces of memory content, others have shown that memory performance could be affected, for instance, by the pattern of eye movements preceding a recognition task (Christman et al., 2003). These findings are in line with successful episodic encoding of neurophysiological data into long-term memory (Guderian et al., 2009) and successful episodic memory retrieval (Addante et al., 2011), and are modulated by preceding brain states of activity reflected as changes in the ongoing oscillatory activity at the theta range (4–8 Hz). The extent to whether eye movements and theta activity could be functionally related remains unknown. Therefore, the combination of measuring eye movement patterns preceding and during a memory task may offer new venues to study the mechanisms and the specific memory content underlying the process of memory success and memory failure.

The possibility to explore reminiscences of memory traces despite participants’ inability to subjectively evaluate the quality or the accessibility of the long-term memories can be seen as an important hallmark in creating new approaches to explore memory functioning ahead of participants’ explicit report or other overt responses. However, some methodological limitations may require further investigation in future experimentation. For instance, even though our findings hold for those trials in which the participant declared not being aware of any type of information related to the memory event (i.e., picture imagery and space location), it is still possible that our design cannot always distinguish information loss from impaired access as a source of forgetting. Thus, it could well be the case that other standard memory tests, e.g., recognition tasks, could enhance the participants’ ability to access memories from long-term through explicit responses. Methodological aspects as such call for further experimentation in the future.

At a broader level, current findings lend support to the notion that the putative systemic division of labor between conscious and unconscious memory is not so clean (Hannula and Greene, 2012). For instance, Voss and Paller (2010) suggested that the relationship between recognition performance and explicit memory might not be so straightforward. Indeed, changes in strategy, based for example on encouragement to guess, can improve recognition performance, but these performance improvements do not always reflect conscious retrieval processes (Voss et al., 2008; Voss and Paller, 2009, 2010). Another example can be seen in the change blindness effect. This effect documents the situation in which the memory representation of scene information and conscious awareness of perceptual changes may not go always together (Simons et al., 2002). In these experiments, participants are unable to consciously detect changes between two scene presentations, although these experiments also show that people often do have a representation of some aspects of the pre-change scene even when they fail to report the change (Simons et al., 2002). Present results contributed to the growing evidence that long-term memory traces can be accessed implicitly. And, in doing so, they challenge the view by which memory systems are essentially divided as to whether they support conscious access to remembered content or not.

In sum, the current results reveal the emergence of a memory-guided behavior that can be used to unconsciously map internal representations of associative memories from long-term memory. They may provide a valuable tool that could open the door to the exploration of, for instance, neurological patients with severe impairments in memory recall and allow the use of comparable paradigms in animals and humans. Future work may put an effort in creating behavioral tasks that could reliably identify memory traces at individual level. While we wait for such advance, they reveal the possibility of investigating memory content reactivation even when explicit (conscious) recollection has failed.

## Conflict of interest statement

The authors declare that the research was conducted in the absence of any commercial or financial relationships that could be construed as a potential conflict of interest.

## Footnotes

^1^http://recursostic.educacion.es/bancoimagenes/web/

^2^www.psychtoolbox.org
